# Hyperthermic intraperitoneal chemotherapy with oxaliplatin as treatment for peritoneal carcinomatosis arising from the appendix and pseudomyxoma peritonei: a survival analysis

**DOI:** 10.1186/1477-7819-12-332

**Published:** 2014-11-07

**Authors:** Eric Marcotte, Pierre Dubé, Pierre Drolet, Andrew Mitchell, Suzanne Frenette, Guy Leblanc, Yves E Leclerc, Lucas Sideris

**Affiliations:** Department of Surgery, Hôpital Maisonneuve-Rosemont, University of Montreal, 5415 boulevard de l’Assomption, Montréal, Québec H1T 2 M4 Canada; Department of Anesthesiology, Hôpital Maisonneuve-Rosemont, University of Montreal, 5415 boulevard de l’Assomption, Montréal, Québec H1T 2 M4 Canada; Department of Pathology, Hôpital Maisonneuve-Rosemont, University of Montreal, 5415 boulevard de l’Assomption, Montréal, Québec H1T 2 M4 Canada; Department of Pharmacy, Hôpital Maisonneuve-Rosemont, University of Montreal, 5415 boulevard de l’Assomption, Montréal, Québec H1T 2 M4 Canada

**Keywords:** Peritoneal neoplasms, Appendiceal neoplasms, Hyperthermic intraperitoneal chemotherapy, Oxaliplatin

## Abstract

**Background:**

Appendiceal peritoneal carcinomatosis (PC) is rare and its long-term prognosis is poor. The aim of this study was to evaluate the results of an aggressive treatment approach used in our institution for the last eight years.

**Methods:**

Data from all patients with PC arising from the appendix were prospectively collected and analyzed. Treatment consisted of complete surgical cytoreduction (CRS), followed by hyperthermic intraperitoneal chemotherapy (HIPEC) with oxaliplatin (460 mg/m^2^) at 43°C over 30 minutes. Ronnett’s histologic classification was used for tumor grading.

**Results:**

Between February 2003 and April 2011, 78 patients underwent laparotomy with curative intent. The mean follow-up period was 33.7 months. A total of 58 patients received HIPEC, but 11 patients could not have CRS and received no HIPEC. Nine patients with a negative second-look surgery also received no HIPEC. The five-year overall survival for the entire cohort was 66.2%; 100% for the negative second-look patients, 77% for the HIPEC patients and 9% for the unresectable patients (*P* <0.0001). A total of 15 patients (25.9%) had isolated peritoneal recurrence, no patient had visceral recurrence only, and five patients (8.6%) had both. In regards to the five-year disease-free survival for the HIPEC patients, histologic grade (disseminated peritoneal adenomucinosis 100%, peritoneal mucinous carcinomatosis with intermediate features 40%, peritoneal mucinous carcinomatosis 20%; *p* =0.0016) and completeness of cytoreduction (CCR-0 56%, CCR-1 24%; *P* =0.0172) were prognostic factors. There was one postoperative mortality. The major complication rate for patients treated with HIPEC was 40%, including intra-abdominal abcess (17%), hemorrhage (12%) and anastomotic leak (10%). One patient in the HIPEC group experienced temporary grade II neuropathy and grade III thrombocytopenia.

**Conclusions:**

This therapeutic approach seems both feasible and safe in selected patients. Recurrence is, however, frequent and represents a challenge.

## Background

Neoplasms of the appendix are rare, with their age-adjusted incidence rate being 0.12 to 2 cases per 1,000,000 people per year [1]. They are found in about 1% of appendectomy specimens [2]. Epithelial tumors, which represent the majority of the appendiceal cancers [1], often present with peritoneal dissemination.

For those cases, the traditional approach has consisted in repetitive debulking surgery and the long-term prognosis (five and ten-year overall survival (OS)) of these patients has been demonstrated in two studies as being 53% and 32% [3], and 65% and 21% [4], respectively. A more aggressive approach consisting of complete surgical cytoreduction (CRS) and intra-peritoneal chemotherapy has been introduced by Gonzalez-Moreno and Sugarbaker in 1983, with five- and ten-year OS rates of 71.9% and 54.5%, respectively [5]. There are many studies published in the last decade similarly showing improvement in long-term survival when compared to traditional debulking surgeries, with five- and ten-year OS ranging from 40 to 87% and 50 to 74%, respectively [6–15]. The addition of hyperthermia to chemotherapeutic agents has been shown to enhance their cytotoxicity [16] and their penetration into tumors [16–18].

There is great variability in regards to the agent used for the intraperitoneal chemotherapy protocol. Some investigators use mitomycin-C or 5-fluorouracil, whereas Elias *et al.* have introduced oxaliplatin as a hyperthermic intraperitoneal chemotherapy (HIPEC) agent for peritoneal carcinomatosis (PC) arising from the appendix [19]. In a phase one study, intraperitoneal oxaliplatin had an advantageous pharmacokinetic profile, displaying very high intraperitoneal concentrations but low systemic toxicity because of limited systemic absorption [20]. Its advantages also include a reduced duration of perfusion, from 90 minutes for mitomycin-C to 30 minutes, as well as a potentialization of peritoneal concentration, while reducing systemic (plasmatic of portal) concentrations in the context of HIPEC, as shown through animal studies performed in our center [18].

The aim of this study was to analyze overall surgical outcome, long-term survival, as well as to identify factors of prognostic value for the patients treated in our institution over the last eight years. It is an update on our series published in 2008 and remains the first to use oxaliplatin exclusively as a cytotoxic agent in HIPEC for PC arising from the appendix.

## Methods

### Patients

From a prospective database, we included all patients (n =78) with peritoneal surface dissemination of epithelial appendiceal tumors treated in our center between February 2003 and February 2011. Our institution’s clinical trial review board approved this study. Hôpital Maisonneuve-Rosemont, University of Montreal. Patients are agreeing to be part of the database when consenting to care. The following factors were analyzed: demographic data, surgical procedures, pathologic diagnosis, complications and length of hospital stay.

Patients were offered treatment consisting of maximal surgical cytoreduction and HIPEC with oxaliplatin if they fulfilled the following criteria: diagnosis proven by histological examination, no evidence of visceral metastasis on computed tomographic (CT) imaging of the chest and abdomen, a technically resectable disease, and a general health status good enough to tolerate the proposed surgery.

### Surgery for peritoneal carcinomatosis and hyperthermic intraperitoneal chemotherapy

The surgical technique for both PC and open-abdomen HIPEC with oxaliplatin (460 mg/m^2^ at 42 to 44°C for 30 minutes) has been previously described [21].

### Postoperative course

Patients were followed up on every day following surgery. Surgical complications were graded using a five-point scale [22]. Minor complications (grade I or II) that were managed with pharmacological treatment (for example urinary tract infection treated with antibiotics) or non-invasive procedures (for example nasogastric tube for postoperative ileus) were not considered. Major complications were defined as grade III to V. A grade III complication is one requiring surgical, endoscopic or radiological intervention (such as an intra-abdominal abscess). A grade IV complication is considered life-threatening and requires ICU management (such as hemorrhagic shock). Grade V is defined as postoperative mortality. We included all complications directly related to the surgical procedure, even if they occurred beyond 30 days postoperatively. Chemotherapy-associated complications were graded according to the Common Terminology Criteria for Adverse Events (CTCAE) version 4.0.

### Pathology

Pathologic classification was performed by an experienced pathologist in our hospital. When surgery of the primary tumor had not been performed in our institution, the pathology material was reviewed by the same pathologist to provide uniform application of diagnostic and grading criteria. Tumor grading of both primary (when available) and peritoneal deposits was done according to Ronnett’s histologic classification [23]. Disseminated peritoneal adenomucinosis (DPAM) was characterized histologically by the presence of scant low-grade adenomatous mucinous epithelium within abundant extracellular mucin and associated fibrosis. Peritoneal mucinous carcinomatosis (PMCA) displayed the cytologic and architectural features of higher-grade mucinous carcinoma associated with extracellular mucin, often with invasive components and sometimes demonstrated signet-ring cell differentiation. Peritoneal mucinous carcinomatosis with intermediate features (PMCA-I) presented with combined DPAM and PMCA characteristics; such tumors were invariably derived from well-differentiated mucinous adenocarcinomas of the appendix.

### Systemic chemotherapy

Perioperative systemic chemotherapy was administered to all PMCA patients. Systemic chemotherapy was also administered preoperatively for three to six months to patients with lower grade disease with extensive disease, defined as an estimated Peritoneal Cancer Index (PCI) [24] greater than 25. This systemic treatment was aimed at diminishing the tumor burden in order to maximize the chance of complete surgical cytoreduction thereafter. Chemotherapy consisted of 5-fluorouracil with irinotecan or oxaliplatin.

### Follow-up

Patients were seen at an outpatient clinic at four-month intervals, at which a physical examination was performed. A CT scan of the abdomen and pelvis was performed every four months for two years, every six months for an additional three years and yearly thereafter.

### Statistics

Data were obtained from a prospective database of clinical records as well as surgical, pathological and radiological reports. No patients were lost during the follow-up period. Kaplan-Meier’s survival curves were established and were compared with log-rank tests. The Cox proportional-hazards regression model was used to analyze the influence of different factors on disease-free survival (DFS) and OS. Differences were considered significant at *P* ≤0.05. Data were analyzed using Prism (GraphPad Software, San Diego, California, United States) and JMP™ 6.0 (SAS Institute Inc. Cary, North Carolina, United States).

## Results

Between February 2003 and February 2011, 78 patients with a PC originating from the appendix underwent laparotomy with curative intent. There were 34 males and 44 females, with a mean age of 50 (range 32 to 70) years. The primary tumor had been removed in 72 patients. At laparotomy, 14 patients were found to have unresectable disease, either because of too extensive PC or intraoperative discovery of visceral metastasis. Of note, two of these patients underwent repeat surgery with successful cytoreduction and HIPEC after six months of systemic chemotherapy. Nine other patients initially diagnosed with a mucinous tumor of the appendix with limited peritoneal disease had no evidence of PC when a second-look laparotomy was performed in our center six to twelve months later. All these patients had undergone appendectomy along with the complete removal of peritoneal tumor in another center before being referred to us. Four of these nine patients with well-differentiated cystadenocarcinoma of the appendix underwent complementary right hemicolectomy at second-look surgery for staging purposes. Since these patients had no evidence of residual disease they were not treated with HIPEC.

Complete surgical cytoreduction followed by HIPEC was performed in 58 patients. The CCR Completeness of Cytoreduction score [25] was 0 for 43 patients and one for 15 patients. In the HIPEC group, the median peritoneal cancer index was 13 (2 to 28), patients had a mean of 1.4 (0 to 5) organs resected and 0.6 (0 to 2) anastomosis. Median operative time was 362 (135 to 855) minutes and the median blood loss was 600 (50 to 7300) ml. The median length of hospital stay for HIPEC patients was 16 (7 to 104) days. One patient died 16 days following surgery and HIPEC. He presented with sepsis and multi-organ failure. The overall major (grade III to V) complication rate was of 39% (23 out of 58), including intra-abdominal abscesses (22%, 13 out of 58), hemorrhage (17%, 10 out of 58) and anastomotic leaks (10% of patients, six out of 58; 17% of anastomoses, six out of 35). One patient in the HIPEC group experienced grade II neuropathy that lasted for one week following surgery. The same patient also developed grade III thrombocytopenia one week postoperatively.

Final pathology reports showed disseminated peritoneal adenomucinosis (DPAM) in 19 patients (24%, 19 out of 78) (HIPEC n =14, negative second-look (on initial pathology) n =5, unresectable n =0), PMCA-I in 41 patients (53%, 41 out of 78) (HIPEC n =34, negative second-look (on initial pathology) n =3, unresectable n =4) and PMCA in 18 patients (23%, 18 out of 78) (HIPEC n =10, negative second-look (on initial pathology) n =1, unresectable n =7).

### Survival rates

**Figure 1:**
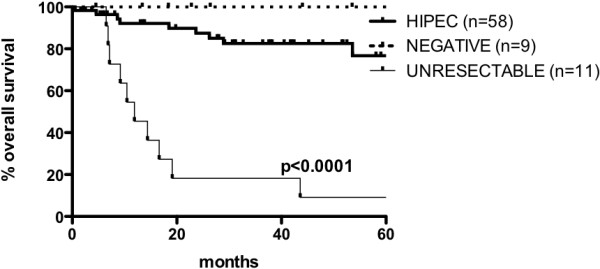
**Overall survival at 60 months for patients who underwent complete surgical cytoreduction followed by hyperthermic intraperitoneal chemotherapy (HIPEC) who were found to have unresectable disease and those who had a negative second-look surgery.**

**Figure 2:**
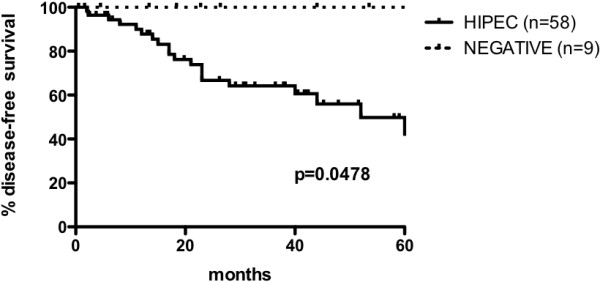
**Disease-free survival at 60 months for patients who underwent hyperthermic intraperitoneal chemotherapy (HIPEC) and those who had a negative second-look surgery.**

The mean follow-up period was 33.7 months (median: 29.1; range: 2 to 100.8) for the entire series. The estimated five-year OS rate for the entire series was 66% (95% CI, 51 to 78). The estimated five-year OS was 100% for the negative second-look patients, 77% (95% CI, 57 to 88) for the HIPEC patients, and 9% (95% CI, 1 to 33) for the unresectable patients (*P* <0.0001) (Figure 1). In the HIPEC group, at the time of data analysis, 15 patients (25.9%) had isolated peritoneal recurrence, no patient had visceral recurrence only, and five patients (8.6%) had both. The estimated five-year DFS for the HIPEC group was 50% (95% CI, 30 to 66) and 100% for the negative second-look group (*P* =0.0478) (Figure 2).

**Figure 3:**
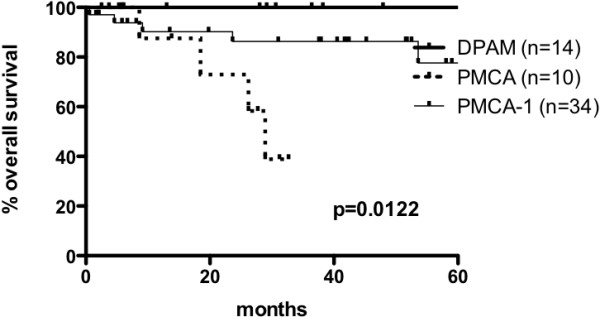
**Overall survival of entire series at 60 months according to histologic grade.** Histologic types: disseminated peritoneal adenomucinosis (DPAM), peritoneal mucinous carcinomatosis (PMCA) and peritoneal mucinous carcinomatosis with intermediate features (PMCA-I).

On univariate analysis, histologic grade was an important prognostic indicator of both five-year DFS for HIPEC patients (*P* =0.0016) and five-year OS for the entire cohort (*P* <0.00001) (Figure 3). The respective five-year DFS and OS was 100% and 100% for DPAM, 40% (95% CI, 17 to 62) and 40% (95% CI, 17 to 62) for PMCA-I, as well as 20% (95% CI, 1 to 55) and 20% (95% CI, 1 to 55) for PMCA. OS curves were statistically different between the PMCA-I and PMCA groups (*P* <0.0003), between the DPAM and the PMCA groups (*P* <0.0003) but they were not statistically significantly different between the DPAM and the PMCA-I groups. The cytoreduction score was also a significant prognostic factor of five-year DFS (*P* =0.0172) with survival of 56% (95% CI, 32 to 74) for CCR-0 and 24% (95% CI, 2 to 61) for CCR-1, but a significant difference in OS was not found between the two groups, with a survival of 81% (95% CI, 56 to 93) for CCR-0 and 64% (95% CI, 32 to 85) for CCR-1. Other parameters such as age, sex, peritoneal index, duration of surgery, blood loss, systemic chemotherapy (neoadjuvant or adjuvant) and the occurrence of major complications had no significant influence on survival at univariate analysis.

Finally, the nine patients who underwent second-look laparotomy and were found to have no disease were also followed and are still disease-free.

## Discussion

PC arising from the appendix is a rare condition with a poor long-term prognosis. An aggressive surgical approach seems to be warranted and many centers have recently published their results. There seems to be a survival benefit of CRS and HIPEC administration compared to surgical debulking alone. For example, Gonzalez-Moreno and Sugarbaker (n =501), Elias *et al.* (n =301), Youssef *et al.* (n =289) and Baratti *et al.* (n =104) reported the largest series of patients treated by CRS and HIPEC, with five-year OS rates of 71.9%, 73%, 87% and 71.9% respectively [5, 10–12] (Table 1). These results compare favorably to the five-year OS rate of 53% by Gough *et al.* and 65% by Miner *et al*., who treated their patients with surgical debulking alone [3, 4].

**Table 1:** **Effectiveness of perioperative intraperitoneal chemotherapy (PIC) for peritoneal carcinomatosis arising from the appendix (pseudomyxoma peritonei)**

Chief-Investigator	PIC	n	Median follow-up (months)	Overall survival (%)				Disease-free survival (%)		
				1 year	3 years	5 years	10 years	1 year	3 years	5 years
Sugarbaker [5]	HIPEC or EPIC (MMC)	501	48	---	---	71.9	54.5	---	---	---
Elias [11]	HIPEC (OX or MMC)	301	88	---	---	73	---	---	---	56
Moran [12]	HIPEC (MMC)	289	39	---	---	87	74	---	---	70
Choudry [15]	HIPEC (MMC)	282	24	---	67.4	52.7	---	---	45.1	32.1
Morris [6]	HIPEC (MMC)	106	23	---	---	75	---	71	51	38
Deraco [10]	HIPEC (MMC)	104	37	---	---	71.9	---	---	---	38.8
Zoetmulder [8]	HIPEC (MMC)	103	51.5	90	70.9	59.5	>50	---	43.6	37.4
Sardi (PMCA) [14]	HIPEC (MMC)	77	18	88	56	40	---	---	---	---
Temple [13]	HIPEC (MMC)	58	28	---	76	62	---	---	48	42
Current study	HIPEC (OX)	58	29.1	---	---	66.2	---	---	---	50

EPIC, early postoperative intraperitoneal chemotherapy; HIPEC, Hyperthermic intraperitoneal chemotherapy; MMC, mitomycin C; OX, oxaliplatin.

Although effective, CRS is a lengthy procedure, with extensive dissection and numerous organ resections in order to achieve maximal cytoreduction (CCR-0 or CCR-1) to allow for the administration of HIPEC. As already published, it was found that completeness of cytoreduction was a prognostic factor for DFS [5, 6, 15]. Although oxaliplatin has been demonstrated to penetrate the peritoneal tissues to a depth of up to 2.5 mm [17, 25], it seems that CRS has a superior impact on prognosis than chemotherapy itself. Therefore, one should attempt maximal efforts to achieve complete macroscopic cytoreduction. However, the capacity to achieve CRS is dependent on the extent of peritoneal disease (PCI score) and grade of tumor [8, 13, 14]. In our series, a CCR-0 resection was achieved in 43 of the 68 patients (63%), consistent with other teams [6, 8, 10, 11, 15]. Median PCI, at 13, is somewhat lower than what was reported by other groups, raising some questioning regarding variability in PCI assessment among different teams.

In regards to tumor grading, there has been recent debate about Ronnett’s original classification into three categories [26]. Indeed, many teams have lately been classifying PC arising from the appendix in two categories (low- versus high-grade) by regrouping PMCA-I lesions with DPAMs because of findings of comparable survival [15, 27]. Our findings that the OS of PMCA-I patients is closer to DPAM than PMCA on univariate analysis support that classification. Others proposed that CP arising from non-mucinous adenocarcinoma (classic type) be regarded as a separate category because they lead to the poorest outcome [28]. Although a binary classification seems to be simpler and more reproducible, until a consensus is established, we have chosen to use the original Ronnett’s classification, in order to compare with other groups and to allow for continuity with the preliminary results we published in 2008 [21].

CRS plus HIPEC remains a procedure with a high rate of major complications (Table 2) [6, 8, 10–12, 14, 15, 29]. However, it is noteworthy that the frequency of systemic (hematologic) toxicity was low, confirming the reports of Elias *et al.* [20]. The fact that chemoperfusion takes 60 minutes less to perform than for the mitomycin-C is another reason why we prefer to use oxaliplatin for HIPEC.

**Table 2:** **Morbidity and mortality of cytoreductive surgery combined with perioperative intraperitoneal chemotherapy (PIC) in peritoneal carcinomatosis arising from the appendix (pseudomyxoma peritonei)**

Chief-Investigator	Center	n	Morbidity (%)	Hematologic toxicity (%)	Mortality (%)
Elias [11]	Multicentric	301	40	20	4.4
Morran [12]	Basingstoke, UK	289	7	2	1.4
Choudry [15]	Pittsburgh	282	24.8	---	1.1
Sugarbaker [29]	Washington, DC	155	27	---	2
Sardi [14]	Baltimore	77	27	---	0
Morris [6]	Sydney	106	49	---	3
Deraco [10]	Milan	104	25	3.8	1
Zoetmulder [8]	Amsterdam	103	54	10.7	11
Current study	Montreal	58	40	1.7	1.7

A total of 15 patients (26%) in the HIPEC group presented an isolated peritoneal recurrence, a median of 18 months after the HIPEC procedure. Five of them were eligible for repeat surgery and accepted to undergo another CRS with HIPEC procedure using mitomycin-C.

It is important to mention that one patient with PMCA disease in the negative second-look surgery in our preliminary series finally received CRS and HIPEC after presenting a peritoneal recurrence 36 months after a negative exploration. He presented with another peritoneal recurrence a year after the HIPEC with oxaliplatin. He then underwent another CRS and HIPEC with mitomycin-C but succumbed to the disease 16 months later. With a median follow-up period of more than two years, the OS and DFS survival for the remaining nine negative second-look patients are both 100%, which gives us cause to believe that a prophylactic HIPEC may not be warranted for patients with low-grade disease.

Elias *et al.* recently published the results of their retrospective trial of prophylactic HIPEC for asymptomatic patients at high risk of presenting colorectal PC (resected synchronous macroscopic PC, ovarian metastasis or tumor perforation) [30]. Peritoneal disease was found in more than half of patients despite negative imaging. All patients (including those without macroscopic evidence of peritoneal disease recurrence) underwent HIPEC with oxaliplatin. OS at five years is encouraging, at 90%. We believe that patients with appendiceal carcinomatosis with high-grade disease (PMCA, classic adenocarcinoma or signet-ring cell lesions) would benefit from a similar approach and receive prophylactic HIPEC.

Concerning patients with unresectable disease, they had a more aggressive disease (64% PMCA and 36% PMCA-I) and 10 out of the 11 patients died a median of 11.2 months after surgery. In a study of 18 patients with peritoneal carcinomatosis arising from an appendiceal adenocarcinoma with signet-ring cells, 10 patients were able to get CRS and HIPEC, whereas eight patients were only administered intravenous chemotherapy because of failure to get complete CRS. Median survival was found to be 27 versus 15 months, respectively [31]. Although not statistically significant (*P* =0.12), it seems that the longer survival supports aggressive surgical management, while being reasonable in order to prevent unwarranted complications. In our series, two patients with bulky disease (PMCA-I) that was deemed unresectable at first laparotomy received intravenous systemic chemotherapy (FOLFOX) followed by abdominal reexploration six months later. They then underwent a CCR-0 cytoreduction and HIPEC. With a follow-up period of 42 and 45 months respectively, they are both alive without evidence of disease.

## Conclusions

This therapeutic approach seems both feasible and safe in selected patients. Survival and morbidity with oxaliplatin is similar to what has been reported for HIPEC with mitomycin-C. The major complication rates of these procedures remain high and constant efforts to reduce the operative morbidity is warranted. Recurrence is, however, frequent and represents a challenge. Chemotherapy is warranted for patients with PC deemed unresectable upon initial exploration and a second-look should be performed since chemotherapy can reduce tumor burden enough to allow for complete cytoreduction and HIPEC.

## Abbreviations

CTCAE: Common Terminology Criteria for Adverse Events
CRS: complete surgical cytoreduction
CCR: completeness of cytoreduction
DFS: disease-free survival
DPAM: disseminated peritoneal adenomucinosis
EPIC: early postoperative intraperitoneal chemotherapy
HIPEC: hyperthermic intraperitoneal chemotherapy
ICU: intensive care unit
MMC: mitomycin C
OS: overall survival
OX: oxaliplatin
PIC: perioperative intraperitoneal chemotherapy
PCI: Peritoneal Cancer Index
PC: peritoneal carcinomatosis
PMCA: peritoneal mucinous carcinomatosis
PMCA-I: peritoneal mucinous carcinomatosis with intermediate features
